# Global survey data on rice breeders' characteristics and willingness to adopt alternative breeding methods

**DOI:** 10.1016/j.dib.2019.103782

**Published:** 2019-02-27

**Authors:** Bert Lenaerts, Bertrand C.Y. Collard, Yann de Mey, Matty Demont

**Affiliations:** a Centre for Environmental Sciences, UHasselt, Hasselt, Belgium; b Agri-food Policy Platform, International Rice Research Institute (IRRI), Los Baños, Philippines; c Department of Earth and Environmental Sciences, KU Leuven, Leuven, Belgium; d Plant Breeding Platform, International Rice Research Institute (IRRI), Los Baños, Philippines; e Business Economics Group, Wageningen University & Research, Wageningen, the Netherlands

## Abstract

The data presented in this article contains information on 189 rice breeders from 51 rice-growing countries around the world. Firstly, this unique dataset permits to lay down a baseline of currently used breeding methods. Secondly, the data allow to make an assessment of the adoption behavior of rice breeders towards alternative breeding methods, and in specific rapid generation advance. A global online survey in Google Forms was conducted to obtain information about the different aspects of the adoption process. Both the raw and cleaned data are made available, along with Stata code to promote further research into adoption of breeding methods by public and private breeding institutes.

**Table:** Specifications table

Subject area	*Economics, Plant breeding*
More specific subject area	*Development economics, agricultural economics, technology adoption*
Type of data	*CSV raw and cleaned data files and Stata do file*
How data was acquired	*Online survey*
Data format	*Raw and cleaned*
Experimental factors	*Survey data of rice breeders in different countries*
Experimental features	*Individual breeder characteristics, internal and external characteristics of the organization and adoption of and willingness to adopt rapid generation advance (RGA)*
Data source location	*Global*
Data accessibility	*Data is available with this article*
Related research article	*Lenaerts, B., Collard, B.C.Y., Demont, M., 2018. Global survey of rice breeders to investigate characteristics and willingness to adopt alternative breeding methods. Agriculture & Food Security 7, 15.*https://doi.org/10.1186/s40066-018-0191-3 *Collard, B.C.Y., Beredo, J.C., Lenaerts, B., Mendoza, R., Santelices, R., Lopena, V., Verdeprado, H., Raghavan, C., Gregorio, G.B., Vial, L., Demont, M., Biswas, P.S., Iftekharuddaula, K.M., Rahman, M.A., Cobb, J.N., Islam, M.R., 2017. Revisiting rice breeding methods – evaluating the use of rapid generation advance (RGA) for routine rice breeding. Plant Production Science 20, 337–352.*https://doi.org/10.1080/1343943X.2017.1391705

**Table:** 

**Value of the data** • Unique data on rice breeding institutes, extending the older and more limited in scope article by Hargrove [1]. • Lays down a baseline of breeding methods used in 2015 for later comparison, valuable for policy evaluation given ongoing transformations in public rice breeding [2], [3]. • Allows an assessment of the adoption behavior of rice breeders towards alternative breeding methods, specifically rapid generation advance (RGA) • The data is relevant to rice breeding professionals and policy makers • The dataset will enable other researchers to construct a more refined adoption framework to be used in follow-up studies

## 1 Data

This dataset contains information on 189 rice breeders from 51 rice-growing countries around the world. The data provided focuses on four aspects of the adoption process: (i) individual breeder characteristics, (ii) internal and (iii) external characteristics of the organization, and (iv) stated reasons for adoption or willingness to adopt rapid generation advance. This information is completed with the current state of adoption or willingness to adopt rapid generation advance for varying levels of intensity (i.e. use as main method, secondary method or for testing only).

## 2 Related research articles

Two research articles are based on this dataset. Lenaerts et al. [6] describe the survey and discuss the variables related to the individual breeder characteristics, the internal and external characteristics of the organization, and the stated reasons for and status of adoption or willingness to adopt rapid generation advance. Collard et al. [3] provide a decomposition by country of the total number of responding breeders using RGA (as main method, secondary method, or for testing only).

## 3 Experimental design, materials, and methods

A global online survey in Google Forms was conducted in 2015 to reach rice breeders worldwide [6]. The individual breeder characteristics, the internal and external characteristics of the organization are recorded for 2015 only. The adoption status of RGA was recorded for an entire breeding career, i.e. adopters may have been using RGA at the time of the survey or may have used it sometime in the past. Given breeding is a continuous process and the survey only captured breeding status in 2015, adoption could have happened at any time in the past (before completion of the survey). The survey was constructed using an adoption framework developed by Lenaerts et al. [6], which in turn was based on England et al. [4] and Rogers [5]. All participants voluntarily consented to participate in the study and were able to withdraw from the survey at any time. Full disclosure was given about the research goals and the privacy of participants was ensured by removing all identifying information within the dataset, such as name, email, and institute's name. Participants were not required to leave identifying information (i.e. possibility to fill in the survey anonymously) [6]. More details about the adoption framework and survey methodology underlying our dataset—including the survey itself—are described in Lenaerts et al. [6]. Stata code to clean the raw data, as well as a legend are available in the Supplementary material to this article.

## Appendix A Supplementary data

Multimedia component 2: Supplementary notes 1 Multimedia component 2

Multimedia component 3: Data file 1 (cleaned) Multimedia component 3

Multimedia component 4: Data file 2 (uncleaned) Multimedia component 4

Multimedia component 5: Stata do-file Multimedia component 5

Multimedia component 6: Supplementary notes 2 Multimedia component 6
