# Supplementary material for: Global survey data on rice breeders' characteristics and willingness to adopt alternative breeding methods
Source: Data Brief. 2019 Feb 27;23:103782. doi: 10.1016/j.dib.2019.103782 (PMC6660548; doi:10.1016/j.dib.2019.103782)
Supplement: Supplementary file 1 — Multimedia component 1: Conflict of Interest Statement [file mmc1.pdf]

Conflict of Interest Statement

Manuscript Title: Global survey data on rice breeders' characteristics and willingness to adopt alternative breeding methods

Corresponding author: Bert Lenaerts (bert.lenaerts@uhasselt.be)

Co- author(s): Bertrand C. Y. Collard (bcycollard@gmail.com), Yann de Mey (yann.demey@wur.nl), Matty Demont (m.demont@irri.org)

The corresponding author confirms on behalf of all authors that:

- All authors have participated in (a) conception and design, or analysis and interpretation of the data; (b) drafting the article or revising it critically for important intellectual content; and (c) approval of the final version.

- This manuscript has not been submitted to, nor is under review at, another journal or other publishing venue.

- The authors have no affiliation or involvement with any organization or entity with a direct or indirect financial or non- financial interest in the subject matter or materials discussed in the manuscript

Printed Name (corresponding author): BERT LENAERTS

Signature (corresponding author):

Date: January 4th, 2019
