# Supplementary material for: Global survey data on rice breeders' characteristics and willingness to adopt alternative breeding methods
Source: Data Brief. 2019 Feb 27;23:103782. doi: 10.1016/j.dib.2019.103782 (PMC6660548; doi:10.1016/j.dib.2019.103782)
Supplement: Multimedia component 6: Supplementary notes 2 [file mmc6.pdf]

Data cleaning guidelines

Bert Lenaerts, Bertrand C. Y. Collard, Yann de Mey,
Matty Demont

1 Import CSV

The raw data (csv-file) that needs to be imported comes directly from the survey as described in the supplementary materials to Lenaerts et al. (2018).

For the variables BUDGET_SIZE and AREA, the comma delimiter needs to be removed before destringing (transform to numerical variable type).

Also one ambiguous observation is removed (WTA_STEP=="A, B, C").

2 Defining Adoption Categories

In a next step, the categorical variable WTA_STEP needs to converted into several adoption variables. Table 1 and Figure 1 give an overview of the different adoption categories into which survey participants can be divided. The first variable, ADOPTION, corresponds to the four categories outlined in Figure 1: no adoption (nothing); adoption for testing, as secondary and as main method (numbered 1 to 4, respectively). This variable is then translated into three binary dummies: RGA_MAIN, RGA_SECOND, and RGA_TESTING. To capture willingness to adopt (WTA), three variables are created: WTA, WTA_base and WTA_using. The variable WTA defines four categories for all participants except those who already adopted as main method: no willingness to adopt any further; willingness to adopt to testing, from testing to secondary and to main method (numbered 1 to 4, correspondingly). The variable WTA_base defines the same four categories as WTA but only for those participants who have not yet adopted RGA as secondary or main method. The variable WTA_using is a binary indicator whether participants use RGA as a main or secondary method.

Table 1. Categories of Adoption and Willingness to Adopt

**Categories**	**Label**	***N***	**Perc.**
No adoption, but WTA for testing	A	24	(15%)
No adoption, but WTA to secondary method	B	42	(27%)
No adoption, but WTA to main method	C	22	(14%)
No adoption nor WTA	D	5	(3%)
Adoption for testing, but WTA to secondary method	E	16	(10%)
Adoption for testing, but WTA to main method	F	5	(3%)
Adoption for testing, but no further WTA	G	1	(1%)
Adoption as secondary method, but WTA to main method	H	26	(16%)
Adoption as secondary method, but no further WTA	I	9	(6%)
Adoption as main method	J	8	(5%)

Notes: 184 observations.

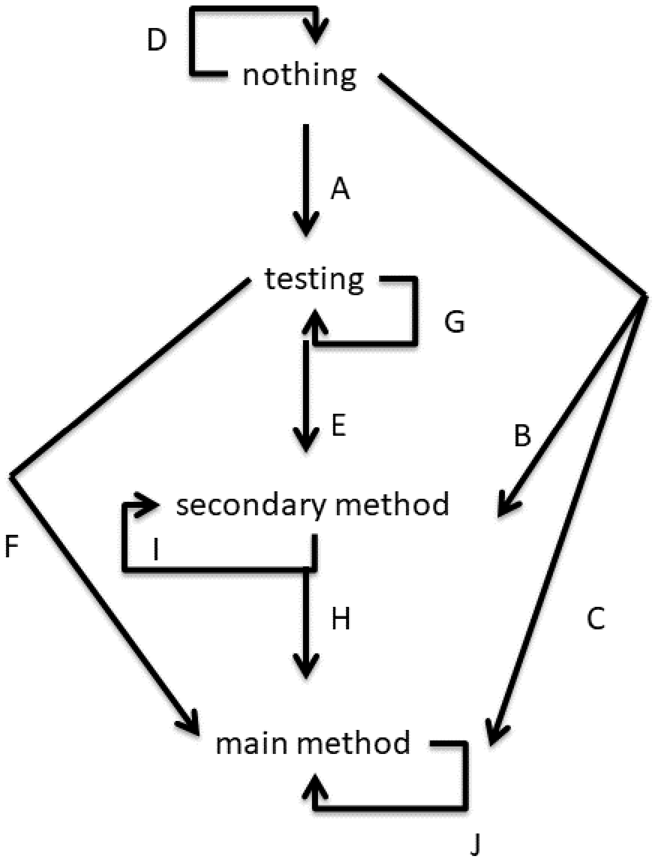

Figure 1. Overview of different adoption and willingness to adopt categories for the variable WTA_STEP.

3 Create Dummies

In this section of the do-file a number of categorical variables are translated into binary indicators. The categories of both the original and new variables can be found in an supplementary Excel-file called 'Legend'.

4 Create Variables

The country-levels of the variable COUNTRY_INSTITUTE were aggregated into (binary) variables for the following continents: Asia, Africa, South America (SA), North America (NA), Europe (including Russia) and the Middle East (ME).

In accordance with Hargrove (1978), we define the set of Western countries to consist of France, the Netherlands, the United Kingdom, Japan, Australia, Canada and the United States.

5 Transformations

This sections transforms some of the original variables into a more convenient form. Firstly, the variable INSTITUTE which is coded 0 for private institutes is replaced by the variable PRIVATE coded 1 for private institutes.

The birth year of each participant is translated into their age at the time of the survey (2015).

The variable FIXED_LINES_CROSSING, which is expressed in number of generations, is transformed into units of years using the average number of growing seasons (FIXED_LINES_CROSSING2) and using the median number of growing seasons (FIXED_LINES_CROSSING3).

Lastly, the variables BUDGET_SIZE and AREA are expressed as 10^4 dollars and m^2, respectively.

References

Hargrove, T.R., 1978. Rice breeders in Asia: a 10-country survey of their backgrounds, attitudes, and use of genetic materials, IRRI Research Paper Series No. 13, February 1978. International Rice Research Institute, Los Baños, Philippines.

Lenaerts, B., Collard, B.C.Y., Demont, M., 2018. Global survey of rice breeders to investigate characteristics and willingness to adopt alternative breeding methods. Agriculture & Food Security 7, 15. https://doi.org/10.1186/s40066-018-0191-3
